# Long-term cultivation alter soil bacterial community in a forest-grassland transition zone

**DOI:** 10.3389/fmicb.2022.1001781

**Published:** 2022-09-29

**Authors:** Zhenyin Bai, Lingbo Zheng, Zhenjian Bai, Aomei Jia, Mingjun Wang

**Affiliations:** Department of Animal Science and Technology, Northeast Agricultural University, Harbin, China

**Keywords:** forest-grass transition zone, grassland, land use, soil properties, soil bacteria

## Abstract

Changes in land use types can significantly affect soil porperties and microbial community composition in many areas. However, the underlying mechanism of shift in bacterial communities link to soil properties is still unclear. In this study, Illumina high-throughput sequencing was used to analyze the changes of soil bacterial communities in different land use types in a forest-grassland transition zone, North China. There are two different land use types: grassland (G) and cultivated land (CL). Meanwhile, cultivated land includes cultivated of 10 years (CL10) or 20 years (CL20). Compared with G, CL decreased soil pH, SOC and TN, and significantly increased soil EC, P and K, and soil properties varied significantly with different cultivation years. Grassland reclamation increases the diversity of bacterial communities, the relative abundance of Proteobacteria, Gemmatimonadetes and Bacteroidetes increased, while that of Actinobacteria, Acidobacteria, Rokubacteria and Verrucomicrobia decreased. However, the relative abundance of Proteobacteria decreased and the relative abundance of Chloroflexi and Nitrospirae increased with the increase of cultivated land years. Mantel test and RDA analysis showed that TP, AP, SOC and EC were the main factors affecting the diversity of composition of bacterial communities. In conclusion, soil properties and bacterial communities were significantly altered after long-term cultivation. This study provides data support for land use and grassland ecological protection in this region.

## Introduction

Soil is one of the most biodiverse habitats on Earth (Fierer and Jackson, 2006; Bender et al., 2016), and soil microorganisms are major components of soil (Roesch et al., 2007), which play an indispensable role in ecosystem function and sustainability (Paul and Clark, 1989; Comer and Perkins, 2020). For example, soil microbes not only drive nutrient and carbon cycles (Kowalchuk and Stephen, 2001; van der Heijden et al., 2008), but also directly and indirectly involved in many other ecosystem services, such as the formation of stable soil aggregates and soil structures to control erosion (Lynch and Bragg, 1985; Rillig and Mummey, 2006). Although there are some functional redundancies in microbial diversity, higher diversity offers a higher possibility for the existence of some alternative species. These alternative species can make up for the loss of function caused by the disappearance of some species under the conditions of temporal and spatial changes, thereby maintaining the function of the ecosystem (Yachi and Loreau, 1999). Meanwhile, a recent study showed that some ecological functions were performed jointly by multiple microorganisms, so functional redundancy may disappear with more functions (Wagg et al., 2019). However, with the decrease of soil microbial diversity, some functions of the ecosystem will be inhibited. For example, reductions in the diversity of methanotrophs (*pmoA* genes) and denitrifiers (*nosZ* 369 genes) could have potential negative consequences for climate regulation on Earth by increasing the amount of methane and N_2_O released to the atmosphere (Trivedi et al., 2019). The diversity of soil microbial community is important in determining soil health (Zhao et al., 2019), which are the foundation of soil function (Emmerling et al., 2002; van der Heijden et al., 2008; Nielsen et al., 2015).

Changes in land use types result from the interaction of human activities and natural factors, which may have important implications for soil ecosystems (Ji et al., 2020). Changes in land use types not only affect soil physical and chemical properties, but also soil biodiversity and soil ecological functions (Lauber et al., 2008; Rasche et al., 2011; Richter et al., 2018). Throughout human history, grasslands have been intensely modified and fragmented by agriculture (Gibson, 2009). According to FAO (2009), global food demand will increase by 70% in the coming decades, which will increase the demand for intensively grown crops (Godfray et al., 2010). Land use intensification from primitive grassland to intensive agricultural systems generally increases soil pH, resulting in decreased soil C content, lower water retention, and poorer soil structure (Finn et al., 2017). For example, Long-term monoculture of cotton or maize in grassland soils significantly decrease soil organic carbon (SOC), macroaggregates, catabolic diversity and microbial biomass (Acosta-Martínez et al., 2010; Kösters et al., 2013). Therefore, it is very important to understand the changes of soil properties and microorganisms after grassland is transformed into cultivated land for monitoring soil quality.

Changes in soil properties (e.g., soil pH, SOC and nutrient content, etc.) can affect the composition and structure of microbial communities (Lauber et al., 2008; Rasche et al., 2011; Richter et al., 2018). Alterations in plant residue and plant root exudation inputs induced by land use changes will affect the soil environmental, and ultimately influence soil microbial community composition and structure (Xu et al., 2021). Agricultural intensification is considered to be one of the biggest threats to global biodiversity (Convention on Biological Diversity, 2010), it has a great impact on soil health and microbial diversity (Degens et al., 2000). For example, Chen et al. (2022) found that conversion of grassland to farmland resulted in a decrease in bacterial biomass. In particular, changes in different tillage regimes can alter plant species and associated soil properties, with lasting effects on soil microbial community composition (Buckley and Schmidt, 2001; Jangid et al., 2008; Yu et al., 2011). For example, biological nitrogen fixation is the most basic physiological process of natural grassland, but the development of intensive agriculture is largely based on industrially produced mineral fertilizers, which leads to simpler soil food webs and fewer functional groups, and reduces overall soil microbial richness (McDaniel et al., 2014; Tsiafouli et al., 2015; Bender et al., 2016). In addition, different kinds of microorganisms have different abilities to deal with various nutrient forms in soil, fertilization will affect their growth competitiveness, thus affecting the diversity, biomass and activity of soil microbial community. Studies have shown that long-term nitrogen application will lead to changes in the overall bacterial community and composition of individual bacterial communities (Rousk et al., 2010), such as Actinobacteria (Jenkins et al., 2009) and Acidobacteria (Zhao et al., 2014). However, the effects of grassland conversion to cultivated land on soil properties and composition and structure of soil microbial community, as well as the main regulators driving the distribution of soil microbial communities across different land use types are still unclear, so understanding the impact of land use change and environmental drivers on soil-microbiome responses is important for soil microbial diversity and soil health.

The forest-grassland transitional zone is a typical ecotone with fertile soil and rich species. Plant community in the transitional zone have a high degree of biodiversity and competitiveness, which are extremely sensitive to environmental changes and have poor ability to resist interference (Anchorena and Cingolani, 2004). Therefore, it is of great significance to understand the impact of human disturbance on the forest and grassland transition zone for the protection and management it. In this study, the mountainous meadow is located in the forest and grassland transition zone between the Great Khingan Mountains and Hulun Buir steppe, which belongs to the semi-humid and semi-arid transition zone with typical transition characteristics of vegetation, soil, land use, climate and other environmental factors, and is a relatively sensitive ecological fragile zone. In the past, many tracts of grassland have been cultivated for years as agricultural land. Therefore, it is necessary to study the impact of long-term cultivation on soil microbial properties. The objectives of this study were to (1) study the effects of long-term cultivation (10-year cultivated land and 20-year cultivated land converted from grassland) on soil properties and the structure, and diversity and composition of soil bacterial communities and (2) clarify the key factors controlling bacterial response to long-term cultivation in Inner Mongolia meadow steppe.

## Materials and methods

### Study site

This study area is located at the western foot of the Great Hinggan Mountains, a forest and grassland transition zone between the Great Khingan Mountains and Hulun Buir Grassland, belonging to Yakeshi City, Inner Mongolia Autonomous Region (49°19´ N, 119°56′ E; 666-680 m asl), which has a cold temperate continental monsoon climate. The annual average precipitation is 350–400 mm, and the precipitation period is mostly concentrated in June to September. The average annual temperature was −1.5°C, the highest and lowest temperatures were 36.2°C and − 48.5°C, respectively. The annual accumulated temperature ≥ 10°C is 1700–2,300°C, the annual average sunshine is 2,511–2,663 h, the frost-free period is about 76–95 days, and the soil type is mainly chernozum soil. The grassland type of the study area is mountain meadow, with rich plant species, high plant community coverage and high grassland productivity. The main grassland plants are *Sanguisorba officinalis*, *Vicia sepium* L., *Lathyrus sativus*, *Carex* spp., etc.

### Experiment design

Three plots in each land use types were selected from the mountain meadows on the outskirts of Yakeshi, Inner Mongolia. The plots were grassland, 10-year cultivated land, and 20-year cultivated land, denoted as G, CL10, and CL20, respectively. The grassland has been mowed for many years without grazing or human improvement. The cultivated lands are continuous tillage, no abandoned in the middle, planting crops mainly for wheat, potatoes, rape, etc., with long-term fertilization. Soil at different depths of 0–10 cm and 10–20 cm (denoted as D10 and D20) was collected.

### Soil sampling and analysis

The samples were collected in July 2021. Three transect lines were set inside G, CL10 and CL20, and three soil sampling points were randomly set on each line. A soil corer (with a diameter of 4 cm) was used to sample the soil with five-point sampling method. Soil samples were taken at different depths of 0–10 cm (D10) and 10–20 cm (D20). Three samples from the same line and the same depth were thoroughly mixed to remove root and soil intrusions, quickly packed into sterile sealed bags, refrigerated in an ice box and quickly transported to the laboratory. The samples were divided into two parts. One part was air-dried, ground and sieved to measure soil physical and chemical properties; other part was stored at −80°C for soil microbial analysis.

### Soil nutrient determination

Soil pH and electrical conductivity (EC; an important indicator of salinity) were determined with soil (air-dried)/water (1:5, w/v) suspensions (Bao, 2008), and soil organic matter (SOM) was assessed by K_2_CrO_7_ volumetric method (External heating method; Sparks et al., 1996). Total nitrogen (TN) and alkali-hydrolyzed nitrogen (AN) were measured by Kjeldahl method and alkali-hydrolyzed diffusion method (Lu, 1999). Phosphorus (P) was extracted by HClO_4_-H_2_SO_4_ method, then added the molybdenum-antimony anti-reagent, colorimetric was performed at 880 nm or 700 nm wavelength, and calculated the total phosphorus content (Bao, 2008). Available phosphorus (AP) was extracted with 0.5 M NaHCO_3_ (pH 8.5), and was determined using molybdenum blue method (UV-752 Shanghai, China; Bao, 2008).

### Soil DNA extraction, Illumina sequencing and data analysis

According to the manufacturer, the total DNA was extracted from 5 g of soil (−80°C) using the TGuide S96 magnetic bead method soil genomic DNA extraction kit [Tiangen Biochemical Technology (Beijing) Co., Ltd., model: DP812]. The total DNA concentration and quality was detected using a microplate reader (manufacturer: GeneCompang Limited, model synergy HTX).

According to the construction of bacterial 16S rRNA amplification library, primers 338F (5’-ACTCCTACGGGAGGCAGCA-3’) and 806R (5’- GGACTACHVGGGTWTCTAAT-3’) were used to amplifies hypervariable regions V3 and V4. PCR amplification was carried out in 0.3 μl Vn F (10 μm; Suzhou Hongxun Biotechnology Co., Ltd.), 0.3 μl Vn R (10 μm; Suzhou Hongxun Biotechnology Co., Ltd.), 5 μl KOD FX Neo Buffer (Beijing Bailing Gram Biotechnology LLC Co., Ltd.), 2 μl dNTP (2 mM each), 0.2 μl KOD FX Neo and 50 ng of template DNA in a 10 μl mixture. The amplification process was pre-denaturation at 95°C for 5 min, 25 cycles of 95°C for 30 s, 50°C annealing for 30 s, 72°C for 40 s, and 72°C for 7 min of extension. Each sample was amplified in triplicate. and PCR products was further purified by VAHTSTM DNA Clean Beads magnetic beads. After purification, agarose with a concentration of 1.8% was used for electrophoresis detection, and the concentration (Qubit) was quantified, and the qualified samples were mixed. The mixed samples were recovered by using the Monarch DNA gel recovery kit, and the quality was checked by the Qsep-400 method. Finally, the Illumina novaseq6000 (novaseq6000, illumina) platform was used for sequencing and microbial community analysis.

Using FLASH (version 1.2.11) software (Magoc and Salzberg, 2011), according to the minimum overlap length of 10 bp and the maximum allowable mismatch ratio in the overlap region of 0.2, the reads of each sample were spliced. In order to obtain high-quality Tag data, we use Trimmomatic (version 0.33) to filter tags whose average quality value is less than 20 and whose length is less than 75% of the Tag length (Bolger et al., 2014). Then split the query sequence into chunks without overlap, and use UCHIME (version 8.1) to compare the database to detect and delete chimeras (Edgar et al., 2011). Sequences were clustered (OTU) at the 97% similarity level, and OTUs were filtered with a threshold of 0.005% of all sequences sequenced using USEARCH (version 10.0; Edgar, 2013). Then used RDP software (Version 2.2) to compare with Silva database, and the bacteria were classified at a confidence interval of 80% (Wang et al., 2007; Quast et al., 2013).

### Statistical analysis

All statistical analyses were performed using R (4.1.2) software. Multivariate analysis of variance was used to analyze the effects of land use type, soil depth and their interactions on soil physical and chemical properties and bacterial communities. In order to calculate the α diversity of each sample, we used the “picante” package and BMKCloud to calculate Chao1, Simpson diversity, and Shannon’s evenness. Two-way ANOVA and TukeyHSD test in the “agricolae” package was used to analyze the significant differences between groups in soil physicochemical properties and soil bacterial α diversity. Spearman rank correlation coefficient was used to calculate the correlation between soil physicochemical properties and soil bacterial α diversity. Based on the “vegdist” package, we calculated the Bray-Curtis distance matrix between OTUs of different samples, analyze the similarity of 999 permutations (ANOSIM), and PCoA visualization was performed on soil bacteria of different sites and depths. The linear discriminant analysis (LDA) effect size (LEfSe) method was used to identify features with significant differences in bacteria between the three sites and different depths by biomarkers, and evaluate the effect size of each feature with a threshold of 4.0 and a significant α of 0.5. To investigate the relationship between soil properties and bacterial communities, we performed RDA analysis of soil properties and bacterial communities using “vegdist” packs and Mantel tests using the “vegan” and “ggcor_master” packages to infer the potential relationship between microbial community composition and measured soil properties. In order to further study, the effect of soil properties on the differences in bacterial community abundance and phylum, order, and genus levels in different sites, Spearman rank correlation coefficient was used.

## Results

### Effects of land use types on soil properties

**Figure 1:**
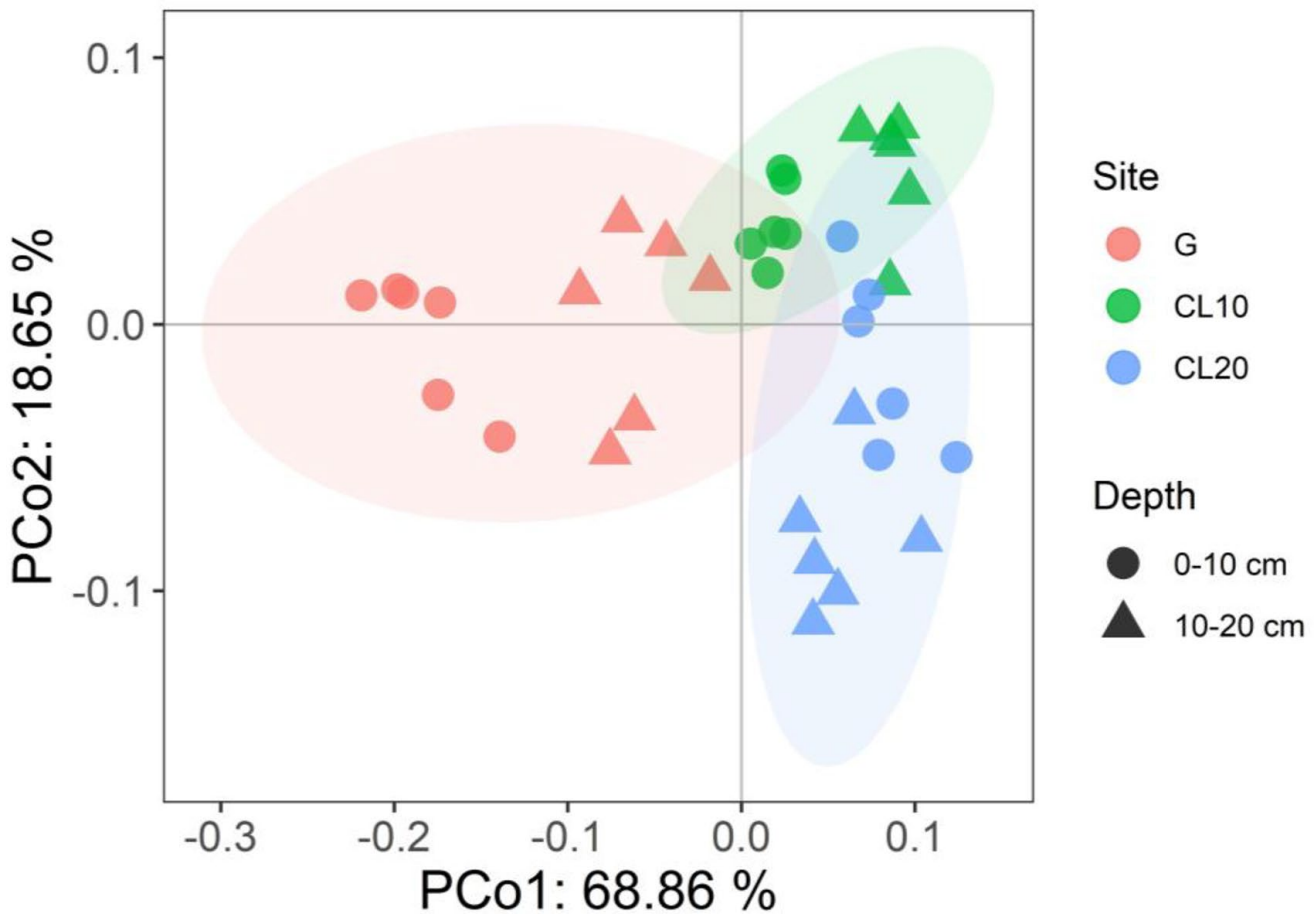
Principal co-ordinates analysis (PCoA) of soil properties among different land use types and depths.

PCoA results showed that soil properties of G, CL10, CL20 and different soil layers were significantly separated, indicating that soil properties of different land use types and different soil layers were significantly different (R^2^ = 0.86299, *p* = 0.001; Figure 1). Different land use types had significant effects on soil properties (*p* < 0.001), soil depth had effects on TP, AP, TK, AK and pH, and years of cultivated land had effects on AN, TP, AP, TK, AK, SOC and EC (Table 1). In the D10 and D20 soil layers, the TN, SOC, pH and C:N of G were the highest (*p* < 0.05). Compared with G and CL10, the contents of AN and TK in each layer of CL20 were the highest (*p* < 0.05). The AN, TP, AP, AK and EC of CL10 were the highest (*p* < 0.05). With the increase of cultivation years, the contents of AN and TK in each soil layer increased (Table 2).

**Table 1:** Anova analysis of the effects of land use type (LUT), soil depth (Depth) and cultivated land years (Year) on soil properties and bacterial diversity and relative abundance.

Effect		LUT	Depth	Year	Effect		LUT	Depth	Year
Soil properties	TN	< 0.001^***^	0.071	0.079	Order level	Myxococcales	0.0122^*^	0.2127	0.587
	AN	0.031^*^	0.207	< 0.001^***^		Xanthomonadales	0.0027^**^	0.096	0.122
	TP	< 0.001^***^	< 0.001^***^	< 0.001^***^		Rokubacteriales	< 0.001^***^	0.085	0.106
	AP	< 0.001^***^	< 0.001^***^	< 0.001^***^		Chthoniobacterales	< 0.001^***^	0.204	0.566
	TK	< 0.001^***^	0.008^**^	< 0.001^***^		Phycisphaerales	0.164	0.922	0.131
	AK	< 0.001^***^	0.0204^*^	0.171		Gemmatimonadales	0.003^**^	0.152	0.520
	SOC	< 0.001^***^	0.129	0.024^*^		Rhizobiales	0.535	0.930	0.186
	pH	< 0.001^***^	< 0.001^***^	0.111		Sphingomonadales	0.004^**^	0.453	0.109
	EC	< 0.001^***^	0.069	0.014^*^		Betaproteobacteriales	< 0.001^***^	0.520	0.076
	C:N	0.001^**^	0.957	0.131		uncultured_bacterium_c_Subgroup_6	< 0.001^***^	0.617	0.038
α diversity	Chao1 richness	0.025^*^	0.997	0.427	Genus level	uncultured_Bacterium_f_SC-I-84	0.037^*^	0.273	0.542
	Simpson diversity	0.071	0.689	0.070		uncultured_Bacterium_f_Burkholderiaceae	0.027^*^	0.786	0.061
	Shannon’s evenness	0.023^*^	0.844	0.159		uncultured_Bacterium_c_KD4-96	0.062	0.603	0.012^*^
	Good’s coverage	0.053	0.5781	0.067		Gemmatimonas	0.085	0.250	0.180
β diversity	Bray-Crutis	< 0.001^***^	0.415	0.422		Candidatus_Udaeobacter	< 0.001^***^	0.229	0.572
Phylum level	Proteobacteria	< 0.001^***^	0.817	0.035^*^		uncultured_Bacterium_o_Rokubacteriales	< 0.001^***^	0.081	0.111
	Acidobacteria	< 0.001^***^	0.462	0.070		uncultured_Bacterium_f_Gemmatimonadaceae	0.001^**^	0.761	0.072
	Actinobacteria	0.001^**^	0.835	0.151		RB41	< 0.001^***^	0.155	0.811
	Chloroflexi	0.020^*^	0.534	0.035^*^		Sphingomonas	0.009^**^	0.409	0.138
	Gemmatimonadetes	0.004^**^	0.118	0.611		uncultured_Bacterium_c_Subgroup_6	< 0.001^***^	0.617	0.038^*^
	Bacteroidetes	< 0.001^***^	0.361	0.056					
	Verrucomicrobia	< 0.001^***^	0.235	0.739					
	Rokubacteria	< 0.001^***^	0.100	0.106					
	Firmicutes	0.032^*^	0.588	0.700					
	Nitrospirae	0.0127^*^	0.153	0.0177^*^					

*(*p* ≤ 0.05); **(*p* ≤ 0.01); and ***(*p* ≤ 0.001).

**Table 2:** Effects of different land use types and depths on soil properties.

Site	TN(g·kg^−1^)	AN(mg·kg^−1^)	TP(g·kg^−1^)	AP(mg·kg^−1^)	TK(g·kg^−1^)	AK(mg·kg^−1^)	SOC(g·kg^−1^)	pH	EC(μS·cm^−1^)	C:N
G-D10	1.81 ± 0.07 b	25.40 ± 2.67 b	0.65 ± 0.18 d	10.20 ± 0.26 d	36.85 ± 6.70 d	115.36 ± 4.59 d	45.54 ± 3.73 a	6.82 ± 0.04 a	46.22 ± 5.84 e	24.99 ± 1.07 a
G-D20	2.07 ± 0.07 a	41.33 ± 2.89 b	1,02 ± 0.32 c	23.90 ± 11.00 c	38.25 ± 5.03 d	153.76 ± 10.81 b	47.57 ± 1.92 a	6.57 ± 0.13 b	58.17 ± 5.69 d	23.04 ± 1.24 ab
CL10-D10	1.61 ± 0.03 c	32.51 ± 2.11 b	1.77 ± 0.12 a	33.26 ± 3.20 b	40.72 ± 3.34 c	153.60 ± 1.77 b	35.63 ± 1.10 b	6.45 ± 0.03 c	81.40 ± 5.22 b	22.18 ± 0.72 b
CL10-D20	1.57 ± 0.04 cd	37.89 ± 7.03 b	1.60 ± 0.17 ab	41.84 ± 1.69 a	43.51 ± 6.09 b	171.55 ± 5.75 a	33.85 ± 0.36 b	6.31 ± 0.09 de	100.58 ± 9.06 a	21.63 ± 0.26 b
CL20-10D10	1.51 ± 0.02 cd	65.92 ± 14.48 a	1.54 ± 0.13 b	29.73 ± 1.38 b	48.45 ± 3.35 b	168.67 ± 1.21 a	28.36 ± 4.73 c	6.37 ± 0.13 cd	75.13 ± 7.79 bc	19.83 ± 3.54 bc
CL20-D20	1.68 ± 0.21 d	44.05 ± 17.20 a	1.34 ± 0.43 ab	31.10 ± 1.94 b	78.87 ± 3.64 a	140.74 ± 1.24 c	35.05 ± 2.18 b	6.24 ± 0.14 e	69.63 ± 18.7 cd	23.32 ± 1.52 c

Values are the means ± SEs (*n* = 3). Different letters represent significant differences between the means (*p* < 0.05).

### Effects of land use types on soil microbial community diversity and composition

A total of 1,440,225 bacterial sequences were obtained after Illumina high-throughput sequencing of 18 samples from 6 groups of samples, and 1,397,395 effective reads were obtained after filtration and removal of low-quality sequences. The effective sequence length was mainly distributed in the range of 400–450 bp, and the average ratio of effective data to original data was 97.03%, with the maximum value 98.7% and the minimum 94.97%. The Q20 of all samples ranged from 98.84 to 99.03%, with an average of 98.95%. There were 31,094 bacterial OTUs in total, and the OTUs of each sample ranged from 1,519 to 2,442, with an average of 1765, and the homology threshold was 97%. The Good’s Coverage index of all samples was higher than 99.18%, indicating that the sequencing quality was good.

**Figure 2:**
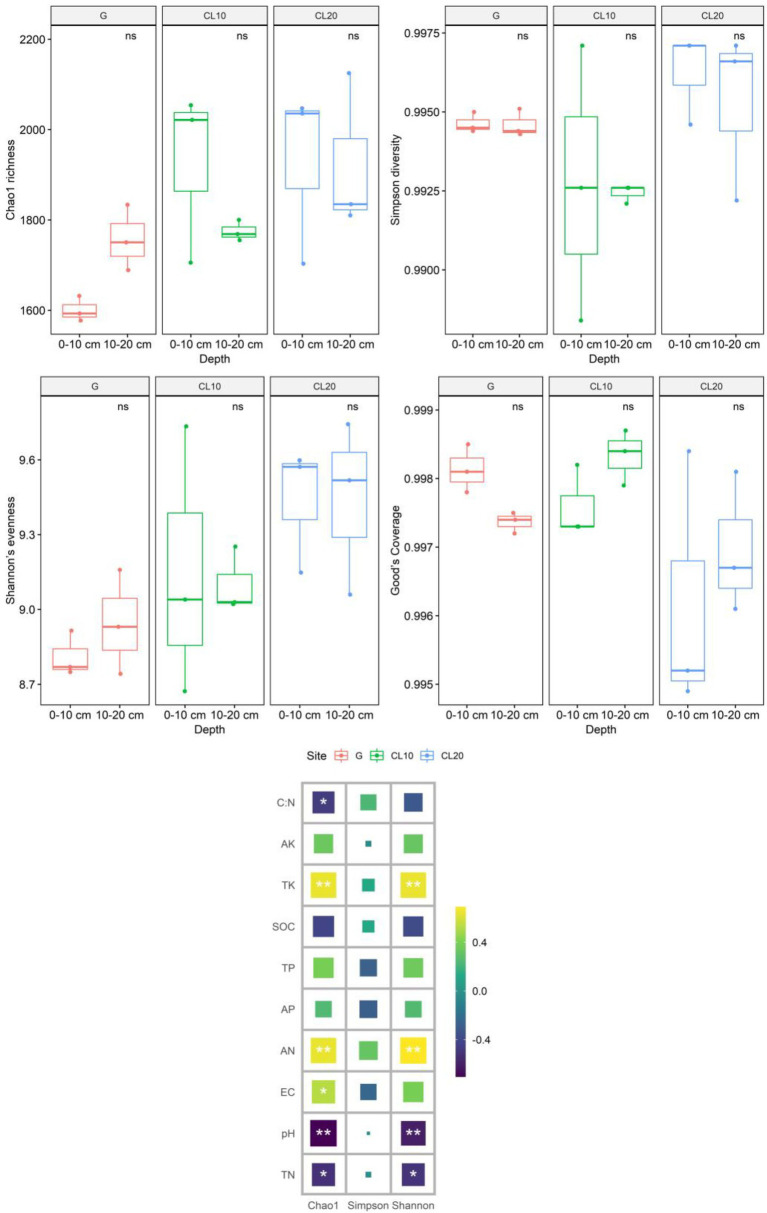
Effects of different land use types, depths, cultivated land years and soil properties on soil bacterial community diversity[Values are the means ± SEs (*n* = 3). Different letters represent significant differences between the means (*p* < 0.05).]

Land use types had significant effects including bacteria Chao1 richness and Shannon’s evenness (*p* < 0.05), but soil depth and years of cultivated land had no effects including Chao1 richness, Simpson diversity, Shannon’s evenness and Good’s coverage (Table 1). Compared with G, the Chao1 richness and Shannon’s evenness in CL were significantly increased, but there were no significant differences among different years of cultivated land. Overall, grassland reclamation increased bacterial richness and evenness, which were not affected by years of cultivated land (Figure 2; Supplementary Table S1). Spearman rank correlation analysis showed that Chao1 richness was positively correlated with AN, EC and TK, and negatively correlated with pH, TN and C:N. Bacterial Shannon’s evenness was positively correlated with AN and TK, and negatively correlated with pH and TN (Figure 2).

**Figure 3:**
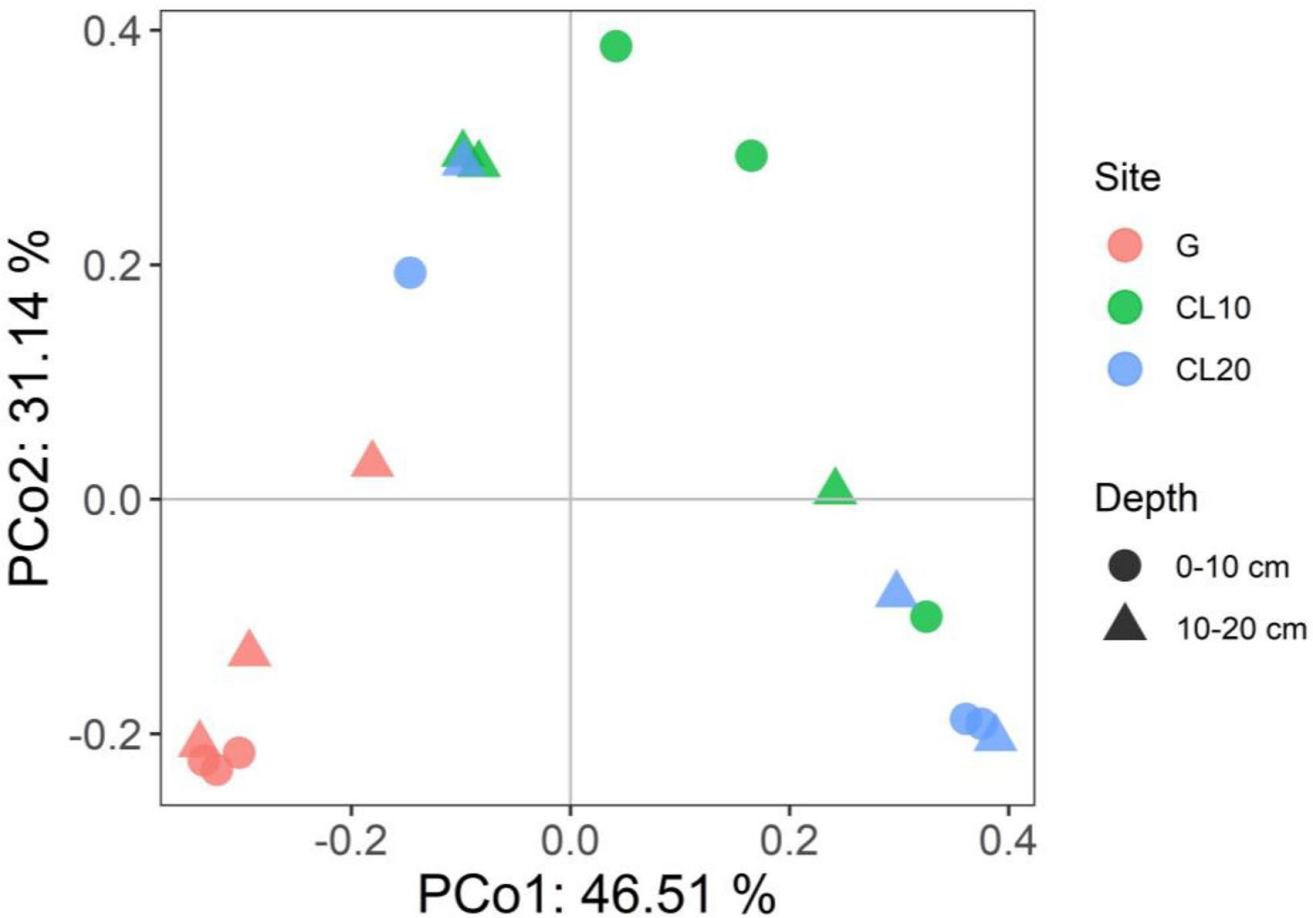
Principal co-ordinates analysis (PCoA) of bacterial communities under different land use types and depths.

Land use type had a significant effect on bacterial *β* diversity (*p* < 0.001), while soil depth and years of cultivated land had no effect on bacterial *β* diversity (Table 1). Based on the Bray-Curtis distance matrix, we performed PCoA visualization of soil bacteria at different land use types and depths. PCo1 and PCo2 explained 46.51 and 31.14% of the variation in bacterial community composition, respectively (Figure 3). The results of Permanova analysis showed that there were significant differences in bacterial community composition among different land use types (R^2^ = 0.55142, *p* = 0.005). Along the directions of PCo1 and PCo2, the bacterial communities in G were far away from those in CL, and the separation of soil bacterial communities between CL10 and CL20 was not significant, indicating that the soil bacterial communities in G and CL were significantly different, and the composition of soil bacterial communities in CL10 and CL20 was similar.

**Figure 4:**
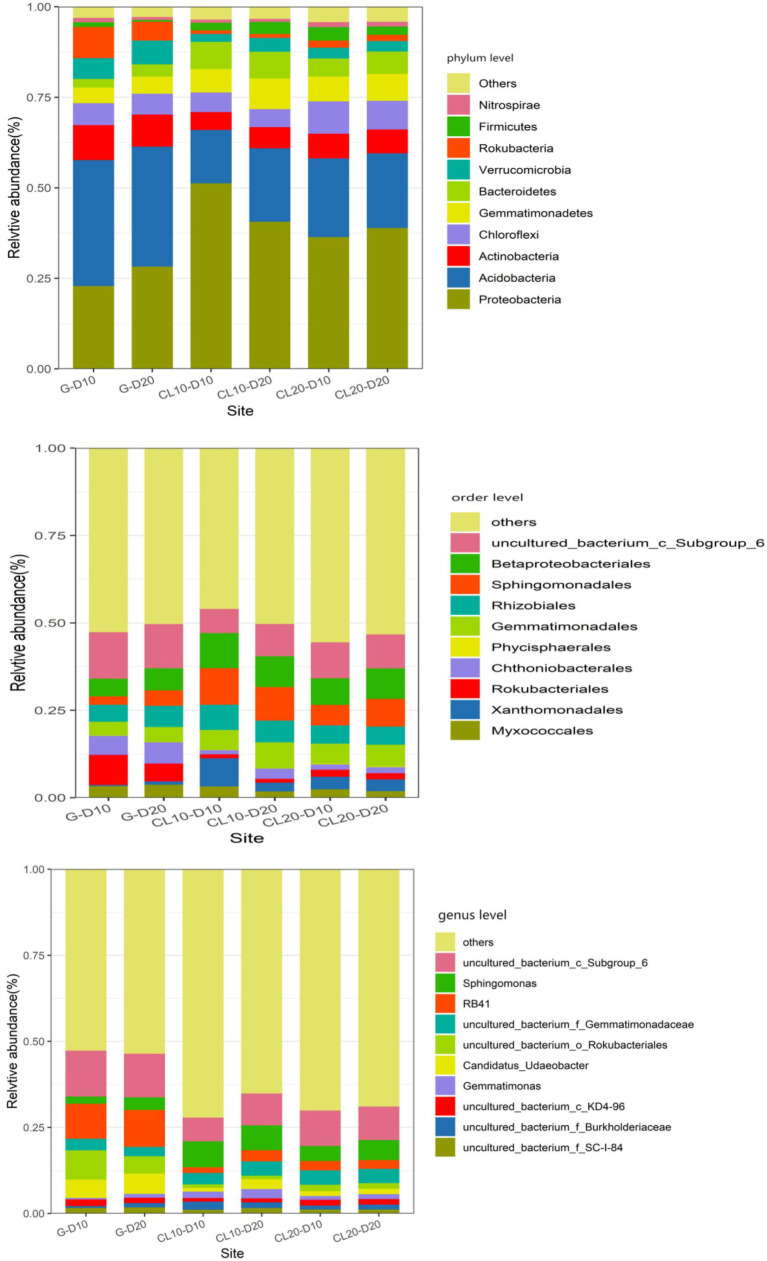
Relative abundance of dominant species in phylum, order and genus level under different land use types and depths.

At the phylum level, land use type had a significant effect on the relative abundance of dominant phylum bacteria, while cultivated land year had a significant effect on the relative abundance of Proteobacteria, Chloroflexi, and Nitrospirae, while soil depth had no effect on the relative abundance of dominant phylum bacteria (Table 1). Soil bacterial phyla in CL10 and CL20 had significantly higher relative abundances of Proteobacteria, Gemmatimonadetes and Bacteroidetes, and significantly lower relative abundance of Actinobacteria, Acidobacteria, Verrucomicrobia and Rokubacteria compared with G. However, the relative abundance of Proteobacteria decreased and the relative abundance of Chloroflexi and Nitrospirae increased with the increase of cultivated land years. Acidobacteria was the most abundant dominant phylum in G, and Proteobacteria was the dominant bacterial phylum in CL10 and CL20 (Figure 4; Supplementary Table S2).

At the order level, except for Phycisphaerales and Rhizobiales, different land use types had significant effects on the relative abundance of other dominant bacteria. Except for the years of cultivated land had significant effects on the relative abundance of uncultured_bacterium_c_Subgroup_6, soil depth and years of cultivated land had no significant effect on the relative abundance of other dominant bacteria (Table 1). The relative abundance of the top 10 dominant bacteria in all soil samples was greater than 48%. The dominant bacteria with the highest abundance in G-D10, G-D20, CL10-D10, CL10-D20, CL10-D10, CL10-D10, CL10-D20, CL20-D10 and CL20-D20 samples were uncultured_bacterium_c_Subgroup_6 (12.61%), uncultured_bacterium_c_Subgroup_6 (13.31%), Sphingomonadales (9.81%), Sphingomonadales (10.95%), uncultured_bacterium_c_Subgroup_6 (9.78%) and uncultured_bacterium_c_Subgroup_6 (10.38%), respectively. The relative abundance of dominant orders was significantly different between CL and G. The relative abundance of Rokubacteriales, Chthoniobacterales and uncultured_bacterium_c_Subgroup_6 in G was higher than that in CL, however, the relative abundances of Xanthomonadales, Sphingomonadales, Gemmatimonadales and Betaproteobacteriales were lower (Figure 4; Supplementary Table S3).

At the genus level, except uncultured_Bacterium_c_KD4-96 and Gemmatimonas, different land use types had significant effects on the relative abundance of other dominant bacteria. The years of cultivated land had significant effect on the relative abundance of uncultured_Bacterium_C_KD4-96 and uncultured_Bacterium_c_Subgroup_6, while the soil depth had no significant effect on the relative abundance of dominant bacteria (Table 1). The relative abundances of the top 10 dominant genera were greater than 28% in all soil samples. The dominant bacteria with the highest abundance in G-D10, G-D20, CL10-D10, CL10-D20, CL20-D10 and CL20-D20 samples were uncultured_bacterium_c_Subgroup_6 (12.61%) and Uncultured_Bacterium_c_Subgroup_6 (13.31%), uncultured_bacterium_c_Subgroup_6 (9.34%), Sphingomonadales (7.87%), uncultured_bacterium_c_Subgroup (9.78%) and uncultured_bacterium_c_Subgroup_6 (10.38%). Compared with G, the relative abundance of uncultured_bacterium_o_Rokubacteriales, Candidatus_Udaeobacter and RB41 in CL soil was significantly decreased. However, the relative abundances of uncultured_Bacterium_f_Burkholderiaceae, uncultured_bacterium_f_Gemmatimonadaceae and Sphingomonas increased significantly. With the increase of cultivated land years, the relative abundance of uncultured_Bacterium_c_KD4-96 and uncultured_Bacterium_c_Subgroup_6 increased significantly (Figure 4; Supplementary Table S4).

**Figure 5:**
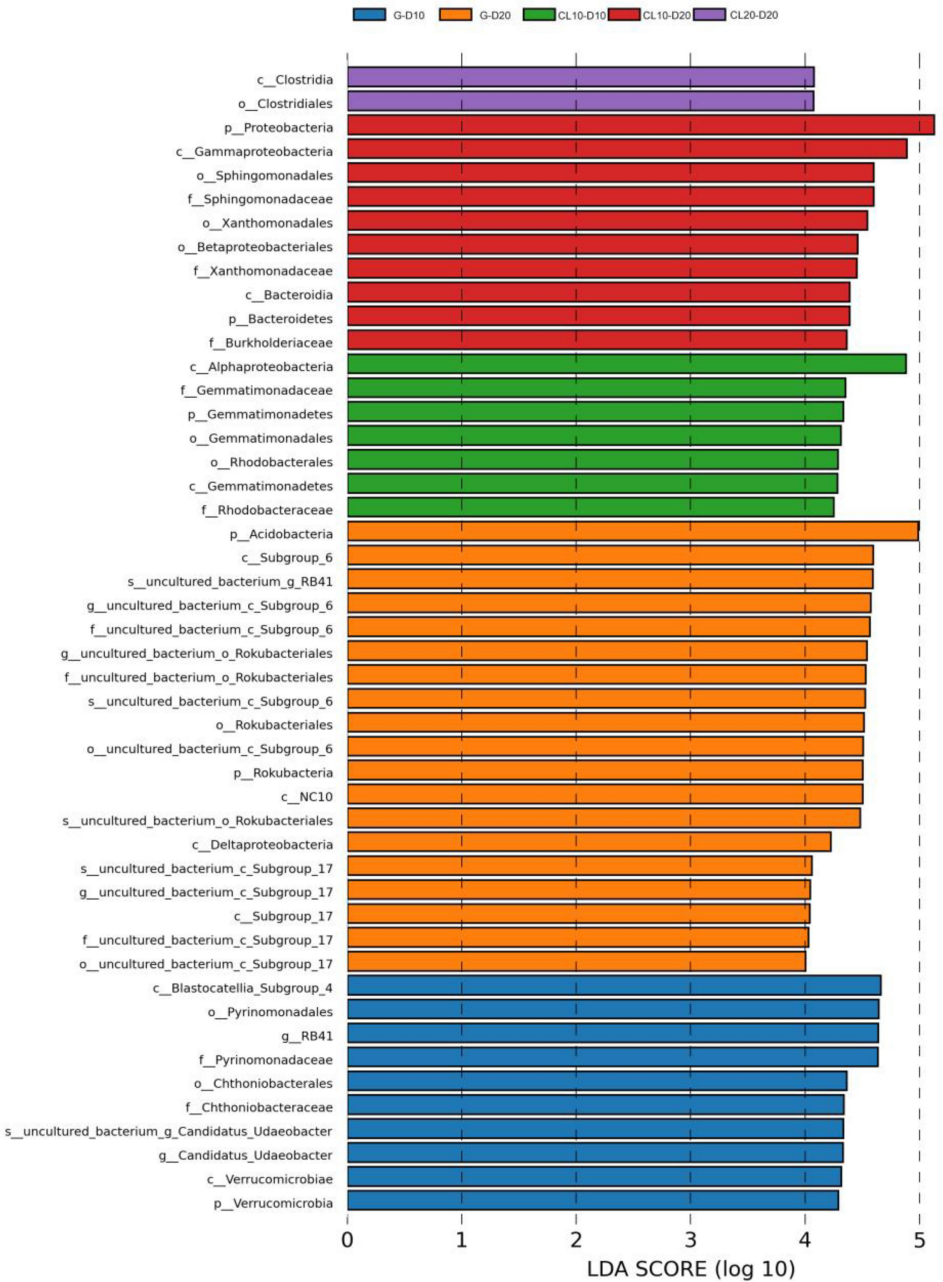
Lefse analysis of bacterial communities under different land use types.

LEfSe was used to identify species with significant differences in abundance in each soil. The results showed that there were 10, 19, 7, 10 and 2 bacterial populations of G-10,G-20,CL10-10, and CL20-20 samples with significant differences, respectively. The species with significant differences in soil abundance were the most abundant in G, indicating that the structure and diversity of bacteria in grassland soil were significantly different from that in CL. It is worth noting that with the increase of cultivated years, the species with significant differences in CL20 decreased significantly, mainly Chloroflexi and Firmicutes (Figure 5).

### Relationships between soil properties and soil bacterial community

**Figure 6:**
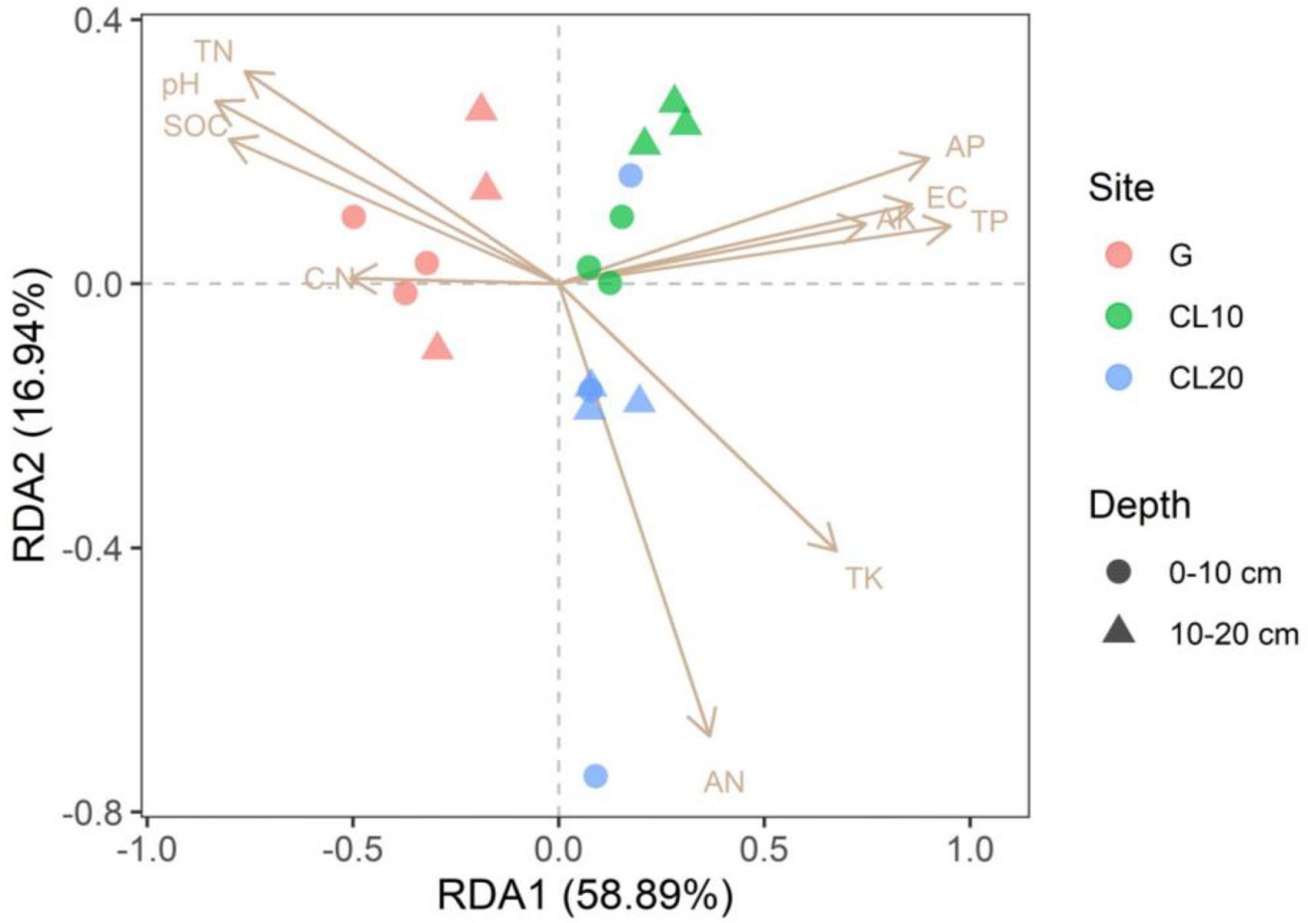
Redundancy analysis (RDA) of bacterial community composition and soil properties in different land use types and depths.

Mantel test was used to analyze the relationship between bacterial community composition and soil properties. The results showed that TP, AP, SOC and EC were the main factors affecting the composition of soil bacterial communities (Table 3). This result was further supported by RDA analysis, with the first RDA1 axis and the RDA1 axis explaining 60.08 and 17.58% of the variation in overall bacterial community composition, respectively. RDA results showed that TN was a soil factor that significantly affected bacterial community composition among all soil traits (*p* = 0.009; Figure 6; Table 4).

**Table 3:** Mantel test of the effects of soil properties on bacteria community composition under different land use types and depths.

	r	*p*
TN	0.305	0.003
AN	0.069	0.192
TP	0.373	8e-04
AP	0.327	0.002
TK	0.271	0.008
AK	0.158	0.044
SOC	0.332	0.002
C:N	0.113	0.105
pH	0.282	0.003
EC	0.341	0.001

**Table 4:** Redundancy analysis (RDA) of the influence of soil properties on bacterial community and bacterial dominant phylum, order and genus levels in different land use types and depths.

	Bacterial community		Phylum level		Order level		Genus level	
	F	*p*	F	*p*	F	*p*	F	*p*
TN	3.655	0.021	9.998	0.005	5.705	0.022	14.353	0.001
AN	0.715	0.589	0.203	0.778	0.412	0.717	0.347	0.729
TP	0.359	0.862	0.045	0.980	0.220	0.835	0.198	0.858
AP	0.662	0.617	0.907	0.374	0.636	0.525	0.872	0.411
TK	0.619	0.648	0.434	0.596	0.481	0.641	0.693	0.473
AK	0.532	0.711	0.366	0.640	0.265	0.799	0.359	0.715
SOC	0.370	0.843	0.281	0.710	0.361	0.723	0.588	0.536
C:N	0.449	0.813	0.502	0.544	0.542	0.591	0.462	0.627
pH	1.330	0.260	1.657	0.236	1.701	0.190	2.688	0.106
EC	1.15	0.339	1.896	0.180	1.135	0.329	1.42	0.254

**Figure 7:**
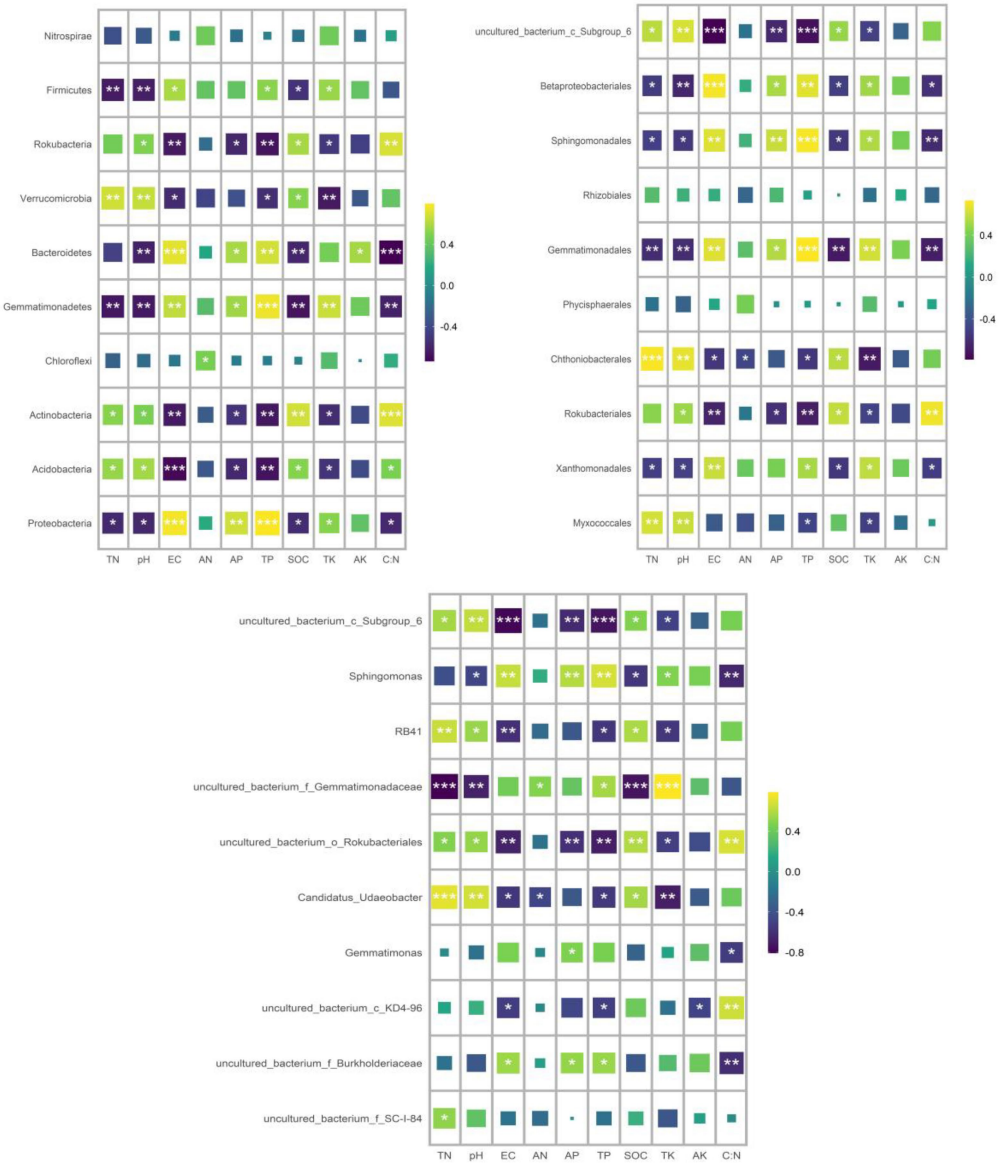
Spearman correlation analysis was conducted to analyze the correlation between the relative abundance of dominant bacterial groups and soil properties.

RDA was used to analyze the relationship between the relative abundance of dominant bacteria at phylum, order and genus level and soil properties. At the phylum level, the first two axes of PDA accounted for 92.01 and 4.13% of the relative bacterial abundance, respectively. At the order level, the first two axes of RDA accounted for 80.75 and 8.35% of the relative bacterial abundance, respectively. At the genus level, the first two axes of RDA accounted for 85.25 and 8.91% of the relative abundance of bacteria, respectively (Supplementary Figure S1). TN was the main factor affecting the relative abundance of dominant bacteria at phylum, order and genus levels (*p* = 0.005, *p* = 0.022, *p* = 0.001; Table 4). Spearman correlation analysis showed that the dominant bacterial phylum Proteobacteria were negatively correlated with TN and pH. Actinobacteria is negatively correlated with AK, EC, AP and TP, while positively correlated with pH. The dominant bacterial order Rokubacteriales were negatively correlated with AK, EC, AP and TP, but positively correlated with pH. Betaproteobacteriales were negatively correlated with EC, AP and TP, but positively correlated with pH. The dominant bacterial genus uncultured_bacterium_c_Subgroup_6 was negatively correlated with EC, AP and TP, but positively correlated with TN, pH and SOC. Sphingomonadales was negatively correlated with AP and TP (Figure 7).

## Discussion

### Effects of land use types on soil properties

Soil properties have high spatial variability among land use types (Jangid et al., 2008; Lauber et al., 2008; Zhang et al., 2020). In our study, we found that the soil properties of G and CL were significantly different. Compared with CL, G had higher SOC content. The reasons for this are, on the one hand, the removal of biomass at harvest time in farmland (Mendez-Millan et al., 2014), and on the other hand, CL is more affected by human activities than grassland, which makes soil loose and plant residues in it easier to decompose (Trivedi et al., 2016). In addition, the conversion from G to CL, due to the extensive use of fertilizers (e.g., urea) and the different uptake of ions by vegetation, crops have a higher uptake of cations, releasing protons (H^+^) into the soil solution and reducing the pH of the soil (Barak et al., 1997; Kunhikrishnan et al., 2016; Flores-Rentería et al., 2020). This acidification process accelerates soil SOC dissolution. However, regardless of CL or G, the SOC content in this study area is relatively higher than that in other areas. It is well known that low temperatures can hinder rapid mineralization of SOC due to the temperature sensitivity of microbial communities (Feng et al., 2017). In the Yakeshi area of Inner Mongolia, the latitude is high, the average annual temperature is only −1.5°C, and the soil temperature is low for a long time. It is decomposed by microorganisms and becomes nutrients that can be directly absorbed and utilized by plant roots. Therefore, the content of SOC in the soil gradually accumulates. We found that compared with G, the contents of N, P and K in CL were higher, and there were no significant differences in different cultivated years (*p* > 0.05). Our results were consistent with previous studies (Ahn et al., 2012; Zhang et al., 2014), which may be attributed to the fact that the heavy application of fertilizers in agricultural management measures (such as long-term use of nitrogen fertilizer) offset the loss of nutrients (Ramirez et al., 2010; Rousk et al., 2010; Smith et al., 2015). In addition, a recent meta-analysis also showed that adding one or more crops in field rotation can effectively increase soil N content (McDaniel et al., 2014). With the increase of cultivated years, soil N, P and K contents increased, but pH decreased. The results were consistent with previous studies (Zhang et al., 2014; Zhou et al., 2015), which may be attributed to the accumulation of nutrients caused by long-term application of chemical fertilizers, which acidified the soil (Guo et al., 2010). In addition, it was found that the increase of soil depth and the decrease of soil pH indicated that the plant roots had a higher uptake of cations and released protons (H^+^) into the soil solution. Kugbe and Issahaku (2015) and Fetene and Amera (2018) also found a strong positive relationship between pH and basic cations. The decrease of basic cations and the increase of H^+^ decreased the pH of the deep soil. The contents of N, P and K in different soil depths were significantly different, which may be due to the reduction of surface litter accumulation by mowing of grassland and harvesting of cultivated plants.

### Effects of land use types on soil microbial diversity and structure

Changes in land use types can directly alter soil physicochemical properties and structure, thereby affecting the diversity of soil microbial communities (Liu et al., 2021). We initially hypothesized that changes in soil properties after grassland reclamation would affect soil microbial community characteristics. Through PCoA analysis, we found that the characteristics of soil bacterial communities were significantly different among the plots. Previous studies have shown that agriculture may reduce soil microbial diversity (Zhang et al., 2020), in contrast, our study found that the richness and diversity of soil bacterial communities increased after grassland reclamation. It is well known that many factors affect soil microbial diversity, such as soil properties (Fierer and Jackson, 2006), land management systems (Carbonetto et al., 2014), and soil vegetation types (Stephan et al., 2000). Soil nutrient limitations in natural grasslands may affect the growth of microorganisms, but the application of fertilizers in agricultural fields creates the possibility for the growth of new species to some extent. At the same time, the regular application of chemical fertilizers in CL also exacerbates the temporal and spatial changes of soil physical and chemical properties, which may also create a certain niche for the growth of microorganisms (Lee-Cruz et al., 2013; Figuerola et al., 2015; Trivedi et al., 2016). In addition, frequent disturbance of the soil environment by certain agricultural practices (such as tillage) in the field actually helps to promote bacterial community diversity and system resilience (Connell, 1978).

Changes in land use types can not only affect microbial diversity, but also effectively shape the structure of soil biota by altering the overall abundance of taxa (Wang et al., 2020). This study found that the abundances of dominant phyla, orders, and genera were significantly different between G and CL, but there was little difference between different cultivated land years. This may be due to the long-term fertilization of cultivated land dominant genera to adapt to the environment. However, both G and CL dominant phyla are Proteobacteria and Acidobacteria, which is consistent with the findings of Zhang et al. (2019). The abundance of Proteobacteria in G was significantly lower than that in CL. Studies have shown that Proteobacteria have various nutritional methods, not only can they maintain their survival through heterotrophic methods, but many special genera can also carry out autotrophic life, such as using photosynthetic pigments to obtain energy from sunlight for metabolism, and energy acquisition methods are diverse. Fertilization allows them to adapt to complex environmental conditions (Mukhopadhya et al., 2012), so soil matrix changes caused by regular fertilization of cultivated land increase the abundance of Proteobacteria in the soil (Goldfarb et al., 2011; Li et al., 2014). Compared with nutrient-rich CL, the abundance of Acidobacteria in G was higher. Acidobacteria is a kind of oligotrophic bacteria, which has unique characteristics of resistance to impoverishment and low temperature, so that it can maintain high metabolic activity under low temperature conditions, the rapid utilization and absorption of OM in the soil. In cold areas, they can rapidly decompose OM by secreting a large number of enzymes related to carbon and nitrogen metabolism (such as β-glucosidase and fibrinose hydrolase, etc.) to obtain energy for their own growth, which is an important dominant bacteria phyla prevalent in cold soil (Yergeau et al., 2010; Peng et al., 2017). In addition, in our study, we found that uncultured_bacterium_c_Subgroup_6 was dominant at the G order and genus level, which is consistent with previous findings (Nacke et al., 2011; Lin et al., 2019). Naether et al. (2012) performed 16S rRNA sequencing on 27 grasslands and 30 woodlands in Germany, indicating that the grassland soils are dominated by the Subgroup_6 subgroup of Acidobacteria. LefSe analysis showed that there were significant differences in the dominant phyla of different land use types, and the species with significant differences in G were significantly higher than those in CL, and the difference became more significant with the increase of cultivated land years. This indicates that the diversity and structure of soil bacteria have been significantly changed after grassland reclamation.

### Effects of soil nutrients on soil microbial community

The richness and diversity of different soil microorganisms are related to specific soil properties (George et al., 2019). Numerous studies have shown that soil pH is related to bacterial community composition (Rousk et al., 2010; Zhao et al., 2014). Our correlation showed that the bacterial richness index was positively correlated with AN and TK, and negatively correlated with pH, TN and C:N. The evenness index was positively correlated with AN and TK, and negatively correlated with pH. Since most bacterial groups exhibit relatively narrow growth tolerance, soil pH may directly influence changes in bacterial communities (Rousk et al., 2010). After grassland reclamation, the contents of N, P and K in CL soil were significantly higher than those in G. Soil properties change is a powerful driver of soil microbial diversity (Fierer, 2017; Tripathi et al., 2018). Therefore, the α-diversity of soil bacteria in CL was significantly higher than that in G. In addition, Mantel test showed that the bacterial community was affected by TN content. Studies showed that soil bacteria were affected by TN content and played a key role in the change of community composition and structure (Thomson et al., 2015; Zhou et al., 2015). Soil TN content can directly affect soil microorganisms, or indirectly affect soil microorganisms by changing soil carbon availability, C:N ratio and soil pH (Treseder, 2008; Serna-Chavez et al., 2013). Therefore, TN content may be second only to pH in influencing soil microbial community composition.

In addition, RDA analysis and Spearman correlation analysis showed that the dominant phylum, order and genus were related to the contents of AK, EC, AP, TP and SOC in soil. Studies have shown that soil P content can affect the relative abundance of different bacteria (Chhabra et al., 2012; Tan et al., 2012). For example, P application reduces soil pH, resulting in a decrease in the abundance of acidic bacteria due to a positive correlation between the abundance of acidic bacteria and soil pH (Jones et al., 2009). At the same time, the increase in soil P content resulted in an increase in the relative abundance of proteobacteria and oligotrophic bacteria in soil (Fierer et al., 2012). In this study, we also found that the relative abundance of some dominant bacteria phyla, orders and genera was negatively correlated with soil salinity (EC), which was consistent with previous studies (van Horn et al., 2014; Xie et al., 2017). With the increase of soil salinity, only a limited number of bacterial groups may be able to withstand the considerable pressure placed on microbial cells in high-salinity soils, leading to changes in the relative abundance of different bacteria and community diversity (Rath et al., 2018). In addition, we also found that the dominant genus uncultured_bacterium_c_Subgroup_6 was positively correlated with SOC, TN, C:N and pH, and negatively correlated with AP and TP. Previous studies have shown that c_Subgroup_6 is relatively abundant in soils with high nutrient levels, and there is a linear positive correlation between soil pH and soil carbon content (Fierer et al., 2007; Jones et al., 2009; Navarrete et al., 2010; Liu et al., 2016). However, Navarrete et al. (2015) and Zhang et al. (2021) showed that c_Subgroup_6 was negatively correlated with soil pH. C_Subgroup_6 correlated positively or negatively with soil pH, suggesting that the same subgroup of Acidobacteria may behave differently in different types of soil (Naether et al., 2012). While SOC is a key resource necessary for most terrestrial microbial communities, SOC levels may affect the abundance of soil bacterial facies and the diversity of bacterial communities (Cai et al., 2016). In summary, our study further demonstrates that different bacteria have different sensitivity to different soil properties, and that soil (microenvironment) heterogeneity is a factor that maintains the diversity of soil microbial communities.

## Conclusion

In conclusion, our study found that the reclamation of mountainous meadow in Inner Mongolia changed soil properties, and with the extension of tillage time, soil nutrients were lost and fertility decreased. Changes in land use types further lead to differences in microbial communities, and there are significant differences in bacterial diversity and community structure between different years. Soil depth had a significant effect on soil properties, but had no effect on soil bacterial communities. Grassland reclamation increases the diversity of bacterial communities, the relative abundance of Proteobacteria, Gemmatimonadetes and Bacteroidetes increased, while that of Actinobacteria, Acidobacteria, Rokubacteria and Verrucomicrobia decreased. And long-term cultivation increased the relative abundance of Chloroflexi and Nitrospirae, while decreased that of Proteobacteria. In addition, we found that the bacterial community is mainly affected by EC, SOC, AP and TP, and different bacteria are affected by different soil properties, which are the factors that maintain the diversity of soil microbial community. Our results provide important information for further understanding soil microbial community changes caused by soil microenvironment changes, which is of great significance for conservation of soil diversity.

## Data availability statement

The datasets presented in this study can be found in online repositories. The names of the repository/repositories and accession number(s) can be found at: NCBI, PRJNA866211.

## Author contributions

ZyB, LZ, and MW conceived and designed this study. ZB drafted the original manuscript. ZjB provided very constructive suggestions for revisions. ZB, LZ, MW, and AJ contributed to the sampling and data analysis. All authors contributed to the article and approved the submitted version.

## Funding

This project was supported by the Special Fund Project for Returnees Studying Abroad of Heilongjiang Province (2021294).

## Conflict of interest

The authors declare that the research was conducted in the absence of any commercial or financial relationships that could be construed as a potential conflict of interest.

## Publisher’s note

All claims expressed in this article are solely those of the authors and do not necessarily represent those of their affiliated organizations, or those of the publisher, the editors and the reviewers. Any product that may be evaluated in this article, or claim that may be made by its manufacturer, is not guaranteed or endorsed by the publisher.

## Supplementary material

SUPPLEMENTARY FIGURE S1 Redundancy analysis (RDA) of bacterial composition and soil properties at dominant phylum, order and genus levels in soils of different land use types and dept.
