# Supplementary figures and images for: Long-term cultivation alter soil bacterial community in a forest-grassland transition zone
Source: Front Microbiol. 2022 Sep 29;13:1001781. doi: 10.3389/fmicb.2022.1001781 (PMC9557053; doi:10.3389/fmicb.2022.1001781)

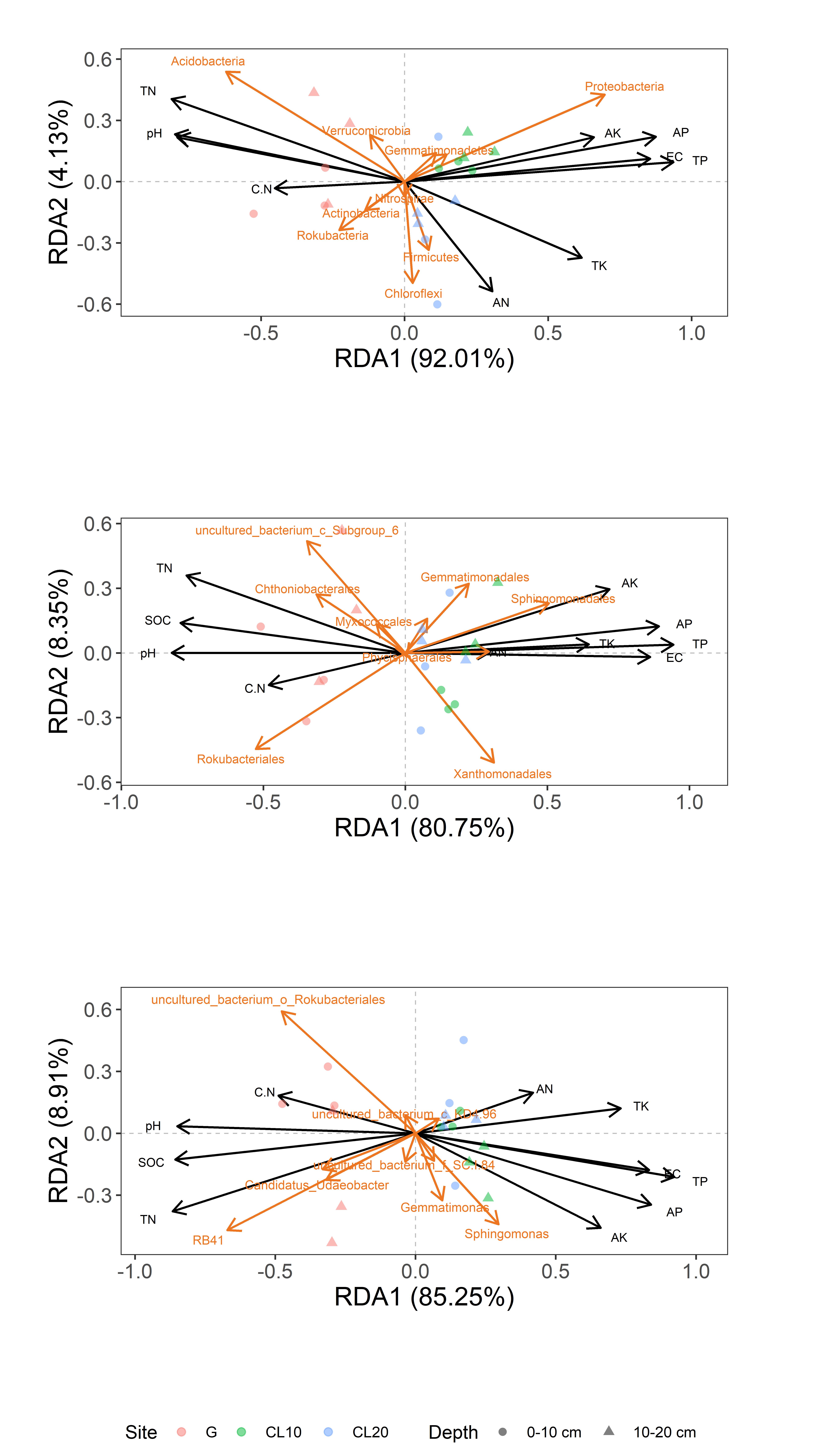

Supplement: SUPPLEMENTARY FIGURE S1 — Redundancy analysis (RDA) of bacterial composition and soil properties at dominant phylum, order and genus levels in soils of different land use types and dept. [file Figure_6.JPEG]
