# Supplementary material for: Long-term cultivation alter soil bacterial community in a forest-grassland transition zone
Source: Front Microbiol. 2022 Sep 29;13:1001781. doi: 10.3389/fmicb.2022.1001781 (PMC9557053; doi:10.3389/fmicb.2022.1001781)
Supplement: Supplementary file 2 [file Data_Sheet_1.PDF]

Table.1 Effects of different land use types and depths on soil properties.Values are the means ± SEs (n=3). Different letters represent significant differences between the means ($P < 0.05$).

Site	TN($g \cdot kg^{-1}$)	AN($mg \cdot kg^{-1}$)	TP($g \cdot kg^{-1}$)	AP($mg \cdot kg^{-1}$)	TK($g \cdot kg^{-1}$)	AK($mg \cdot kg^{-1}$)	SOC($g \cdot kg^{-1}$)	pH	EC($\mu S \cdot cm^{-1}$)	C:N
G-D10	1.81±0.07 b	25.40±2.67 c	0.65±0.18 c	10.20±0.26 c	36.85±6.70 c	115.36±4.59 c	45.54±3.73 a	6.82±0.04 a	46.22±5.84 d	24.99±1.07 a
G-D20	2.07±0.07 a	41.33±2.89 bc	1,02±0.32 bc	23.90±11.00 b	38.25±5.03 c	153.76±10.81 b	47.57±1.92 a	6.57±0.13 b	58.17±5.69 cd	23.04±1.24 a
CL10-D10	1.61±0.03 c	32.51±2.11 c	1.77±0.12 a	33.26±3.20 ab	40.72±3.34 bc	153.60±1.77 b	35.63±1.10 b	6.45±0.03 bc	81.40±5.22 b	22.18±0.72 ab
CL10-D20	1.57±0.04 c	37.89±7.03 bc	1.60±0.17 a	41.84±1.69 a	43.51±6.09 bc	171.55±5.75 a	33.85±0.36 b	6.31±0.09 cd	100.58±9.06 a	21.63±0.26 ab
CL20-10D10	1.51±0.02 c	65.92±14.48 ab	1.54±0.13 ab	29.73±1.38 b	48.45±3.35 b	168..67±1.21 a	28.36±4.73 c	6.37±0.13 cd	75.13±7.79 bc	19.83±3.54 b
CL20-D20	1.68±0.21 c	44.05±17.20 a	1.34±0.43 a	31.10±1.94 b	78.87±3.64 a	140.74±1.24 b	35.05±2.18 b	6.24±0.14 d	69.63±18.7 bc	23.32±1.52 a

Table.2 Effects of different land use types and depths on soil bacterial community diversity. Values are the means ± SEs (n=3). Different letters represent significant differences between the means ($P < 0.05$).

Site	Chao1 richness	Simpson diversity	Shannon’s evenness	Good’s Coverage
G-D10	1600.95±28.11 b	0.99±0.00 a	8.81±0.09 b	99.81±0.04 a
G-D20	1757.87±72.60 ab	0.99±0.00 a	8.94±0.21 ab	99.74±0.02 a
CL10-D10	1927.21±192.40 a	0.99±0.00 a	9.15±0.54 ab	99.76±0.05 a
CL10-D20	1774.84±22.98 ab	0.99±0.00 a	9.10±0.13 ab	99.80±0.04 a
CL20-D10	1928.75±195.45 a	1.00±0.00 a	9.44±0.25 a	99.62±0.19 a
CL20-D20	1923.43±175.11 a	1.00±0.00 a	9.44±0.35 a	99.70±0.10 a

Table.3 Relative abundance differences of dominant species at the Phylum level under different land use types and depths (Values are the means ± SEs (n=3). Different letters represent significant differences between the means (P < 0.05).)

Site	Proteobacteria	Acidobacteria	Actinobacteria	Chloroflexi	Gemmatimonadetes	Bacteroidetes	Verrucomicrobia	Rokubacteria	Firmicutes	Nitrospirae	other
G-D10	0.228±0.006 c	0.348±0.007 a	0.097±0.006 a	0.060±0.002 a	0.043±0.002 b	0.024±0.002 b	0.057±0.008 ab	0.086±0.004 a	0.013±0.009 a	0.012±0.002 a	0.012±0.002 a
G-D20	0.284±0.046 bc	0.331±0.006 a	0.088±0.011 ab	0.058±0.004 a	0.048±0.012 b	0.034±0.015 b	0.065±0.019 a	0.051±0.033 ab	0.005±0.001 a	0.008±0.001 a	0.008±0.001 a
CL10-D10	0.502±0.105 a	0.150±0.033 c	0.052±0.024 b	0.057±0.004 a	0.064±0.009 ab	0.074±0.010 a	0.021±0.004 b	0.011±0.011 b	0.022±0.010 a	0.009±0.005 a	0.009±0.005 a
CL10-D20	0.408±0.022 ab	0.201±0.032 bc	0.058±0.003 b	0.050±0.009 a	0.083±0.017 a	0.073±0.018 a	0.038±0.007 ab	0.011±0.003 b	0.035±0.022 a	0.008±0.002 a	0.008±0.002 a
CL20-D10	0.363±0.005 b	0.213±0.029 b	0.070±0.010 ab	0.091±0.015 a	0.067±0.015 ab	0.050±0.004 ab	0.028±0.017 b	0.020±0.007 b	0.040±0.022 a	0.014±0.001 a	0.014±0.001 a
CL20-D20	0.386±0.021 ab	0.205±0.009 bc	0.068±0.015 ab	0.084±0.031 a	0.072±0.013 ab	0.059±0.020 ab	0.027±0.015 b	0.018±0.007 b	0.025±0.009 a	0.013±0.002 a	0.013±0.002 a

Table.4 Relative abundance differences of dominant species at the order level under different land use types and depths. Values are the means ± SEs (n=3). Different letters represent significant differences between the means ($P < 0.05$).

Site	f_SC-I-84	f_Burkholderiaceae	c_KD4-96	Gemmatimonas	Candidatus_Udaeobacter	o_Rokubacteriales	f_Gemmatimonadaceae	RB41	Sphingomonas	c_Subgroup_6	other
G-D10	0.016±0.001 a	0.005±0.001 b	0.019±0.001 a	0.004±0.000 a	0.054±0.010 ab	0.085±0.004 a	0.034±0.002 ab	0.102±0.003 a	0.021±0.000 a	0.133±0.002 a	0.473±0.020 a
G-D20	0.018±0.001 a	0.012±0.007 ab	0.016±0.005 ab	0.011±0.009 a	0.059±0.021 a	0.050±0.033 ab	0.027±0.005 b	0.107±0.006 a	0.037±0.015 a	0.126±0.009 ab	0.464±0.026 a
CL10-D10	0.011±0.002 a	0.024±0.009 a	0.010±0.003 b	0.019±0.012 a	0.011±0.005 c	0.010±0.010 bc	0.033±0.005 ab	0.017±0.003 b	0.075±0.032 a	0.058±0.022 d	0.278±0.033 b
CL10-D20	0.015±0.004 a	0.017±0.004 ab	0.012±0.001 ab	0.027±0.010 a	0.028±0.007 ab	0.010±0.003 c	0.042±0.004 a	0.033±0.013 b	0.072±0.023 a	0.092±0.014 cd	0.348±0.073 ab
CL20-D10	0.011±0.007 a	0.012±0.002 ab	0.017±0.000 ab	0.011±0.012 a	0.014±0.019 c	0.018±0.006 bc	0.0420±0.004 a	0.027±0.014 b	0.044±0.010 a	0.103±0.007 abc	0.299±0.069 b
CL20-D20	0.011±0.005 a	0.015±0.005 ab	0.016±0.004 ab	0.015±0.014 a	0.016±0.017 bc	0.016±0.006 bc	0.0416±0.001 a	0.025±0.010 b	0.058±0.025 a	0.097±0.002 bcd	0.311±0.066 b

Table.5 Relative abundance differences of dominant species at the genus level under different land use types and depths. Values are the means ± SEs (n=3). Different letters represent significant differences between the means ($P < 0.05$)

Site	Myxococcales	Xanthomonadales	Rokubacteriales	Chthoniobacterales	Phycisphaerales	Gemmatimonadales	Rhizobiales	Sphingomonadales	Betaproteobacteriales	c_Subgroup_6	other
G-D10	0.033±0.001 a	0.003±0.001 b	0.086±0.004 a	0.054±0.010 ab	0.001±0.000 a	0.040±0.002 b	0.048±0.003 a	0.024±0.001 b	0.050±0.004 c	0.133±0.002 a	0.527±0.013 a
G-D20	0.037±0.004 a	0.009±0.006 b	0.051±0.033 ab	0.061±0.020 a	0.001±0.000 a	0.043±0.009 ab	0.061±0.008 a	0.044±0.021 ab	0.063±0.014 bc	0.126±0.009 ab	0.503±0.010 a
CL10-D10	0.032±0.014 a	0.080±0.037 a	0.011±0.011 b	0.012±0.004 c	0.001±0.001 a	0.056±0.010 ab	0.072±0.025 a	0.105±0.042 a	0.100±0.009 a	0.069±0.022 d	0.460±0.100 a
CL10-D20	0.018±0.004 a	0.025±0.003 b	0.011±0.003 b	0.029±0.007 abc	0.001±0.001 a	0.074±0.015 a	0.062±0.013 a	0.096±0.030 ab	0.088±0.016 ab	0.092±0.014 cd	0.503±0.094 a
CL20-D10	0.024±0.014 a	0.036±0.002 b	0.020±0.007 b	0.015±0.019 c	0.002±0.001 a	0.057±0.016 ab	0.052±0.018 a	0.059±0.009 ab	0.076±0.008 abc	0.103±0.007 abc	0.556±0.068 a
CL20-D20	0.019±0.004 a	0.033±0.002 b	0.018±0.007 b	0.017±0.017 bc	0.002±0.002 a	0.062±0.015 ab	0.052±0.014 a	0.079±0.030 ab	0.087±0.006 ab	0.097±0.002 bcd	0.533±0.079 a

Table.6 Mantel test of the effects of soil properties on bacteria community composition under different land use types and depths

	r	*p*
TN	0.305	0.003
AN	0.069	0.192
TP	0.373	8e-04
AP	0.327	0.002
TK	0.271	0.008
AK	0.158	0.044
SOC	0.332	0.002
C:N	0.113	0.105
pH	0.282	0.003
EC	0.341	0.001

Table.7 Redundancy analysis (RDA) of the influence of soil properties on bacterial community composition in different land use types and depths

	TN	pH	EC	AN	AP	TP	SOC	TK	AK	C:N
p	0.025**	0.264	0.342	0.572	0.621	0.872	0.836	0.656	0.709	0.771

Table.8 Redundancy analysis (RDA) of the influence of soil properties on bacterial dominant phylum, order and genus levels in different land use types and depths

	phylum		order		genus	
	F	*p*	F	*p*	F	*p*
TN	9.998	0.005	5.705	0.022	14.353	0.001
AN	0.203	0.778	0.412	0.717	0.347	0.729
TP	0.045	0.980	0.220	0.835	0.198	0.858
AP	0.907	0.374	0.636	0.525	0.872	0.411
TK	0.434	0.596	0.481	0.641	0.693	0.473
AK	0.366	0.640	0.265	0.799	0.359	0.715
SOC	0.281	0.710	0.361	0.723	0.588	0.536
C:N	0.502	0.544	0.542	0.591	0.462	0.627
pH	1.657	0.236	1.701	0.190	2.688	0.106
EC	1.896	0.180	1.135	0.329	1.42	0.254
